# Tissue Factor promotes breast cancer stem cell activity *in vitro*

**DOI:** 10.18632/oncotarget.13928

**Published:** 2016-12-13

**Authors:** Hudhaifah Shaker, Hannah Harrison, Robert Clarke, Goran Landberg, Nigel J. Bundred, Henri H. Versteeg, Cliona C. Kirwan

**Affiliations:** ^1^ The University of Manchester, Manchester Academic Health Science Centre, Department of Academic Surgery, University Hospital of South Manchester, Manchester, UK; ^2^ Faculty of Life Sciences, University of Manchester, Manchester, UK; ^3^ Breast Biology Group, Manchester Cancer Research Centre, University of Manchester, Manchester, UK; ^4^ Sahlgrenska Cancer Center, University of Gothenburg, Sweden; ^5^ Department of Thrombosis and Hemostasis, Einthoven Laboratory for Experimental Vascular Medicine, Leiden University Medical Center, Leiden, Netherlands

**Keywords:** cancer stem cells, tissue factor, breast cancer

## Abstract

Cancer stem cells (CSCs) are a subpopulation of cells that can self-renew and initiate tumours. The clotting-initiating protein Tissue Factor (TF) promotes metastasis and may be overexpressed in cancer cells with increased CSC activity. We sought to determine whether TF promotes breast CSC activity in vitro using human breast cancer cell lines. TF expression was compared in anoikis-resistant (CSC-enriched) and unselected cells. In cells sorted into of TF-expressing and TF-negative (FACS), and in cells transfected to knockdown TF (siRNA) and overexpress TF (cDNA), CSC activity was compared by (i) mammosphere forming efficiency (MFE) (ii) holoclone colony formation (Hc) and (iii) ALDH1 activity. TF expression was increased in anoikis-resistant and high ALDH1-activity T47D cells compared to unselected cells. FACS sorted TF-expressing T47Ds and TF-overexpressing MCF7s had increased CSC activity compared to TF-low cells. TF siRNA cells (MDAMB231, T47D) had reduced CSC activity compared to control cells. FVIIa increased MFE and ALDH1 in a dose-dependent manner (MDAMB231, T47D). The effects of FVIIa on MFE were abrogated by TF siRNA (T47D). Breast CSCs (*in vitro*) demonstrate increased activity when selected for high TF expression, when induced to overexpress TF, and when stimulated (with FVIIa). Targeting the TF pathway *in vivo* may abrogate CSC activity.

## INTRODUCTION

Breast cancer is the most common cancer in women in the UK resulting in over 11,000 deaths per year [1]. Advances in local treatment (surgery, radiotherapy) and adjuvant therapy have increased survival in early breast cancer, however almost a fifth of patients will develop local or distant recurrence within 5 years of diagnosis [2]. Cancer recurrence and subsequent death from metastasis may occur due to a subpopulation of cancer cells known as cancer stem cells (CSC), also known as cancer initiating cells or cancer stem-like cells. CSCs have the ability to initiate tumours and, in a manner similar to normal tissue stem cells, are able to self-renew and replicate into the heterogeneous population [3]. Existence of this CSC subpopulation has been demonstrated in patient-derived cancers [4] and in human breast cancer cell lines [5]. Due to their relative resistance to radiotherapy and chemotherapy, CSCs are believed to be responsible for tumour relapse and represent an important therapeutic target [6]

Experimentally, CSC activity is determined by the assessment of *in vivo* tumour initiation in xenograft models [7]. Cost-effective *in vitro* assays have been developed that act as reliable surrogate markers of CSC activity. The best described is the tumoursphere assay (known as the mammosphere assay in breast cancer) which relies on the inherent resistance of CSC to apoptosis in the absence of normal adherence (known as anoikis). ‘Anoikis-resistant’ cells form floating colonies (mammospheres) when grown in non-adherent culture [8]. Mammosphere formation acts as surrogate marker of *in vivo* tumour formation. Similarly, when grown in adherent culture at extremely low density, cancer cells form three distinct colonies; holoclones, meroclones and paraclones. Holoclone colony formation, which enriches for CSC, is also a well-established CSC activity assay [9]. In addition, stem cell markers have been identified that enrich for CSCs. Enzymatic activity of the cytosolic protein enzyme ALDH1, for example, acts as a marker to enrich for CSCs as well as a marker of increased CSC activity [5].

Tissue Factor (TF) is a multi-functional transmembrane protein whose primary role is initiation of the extrinsic clotting pathway [10]. TF is overexpressed in several cancers and its expression correlates with advanced stage and reduced survival [11]. Cancer-associated dysregulation of TF is well described in pre-clinical studies in which cell membrane expression of TF is upregulated in malignant transformed cell lines [12] and contributes to apoptosis resistance and metastasis [13]. TF also promotes anoikis resistance [14] and is upregulated in the presence of epithelial to mesenchymal transition (EMT) [15]. Both anoikis resistance and EMT are characteristic features of CSC function [16] [17]. One study has demonstrated TF upregulation in association with the CSC marker CD133 [18], however limited studies have examined TFs direct role in breast or any other CSCs.

Here we demonstrate *in vitro* that breast cancer stem cells derived from cancer cell lines demonstrated increased activity when TF expression or activity is modulated. This has therapeutic implications for tumours and treatment of breast cancers by targeting TF and reducing recurrence by killing CSCs.

## RESULTS

### Tissue Factor is upregulated in CSC-enriched T47D cancer cells

**Figure 1:**
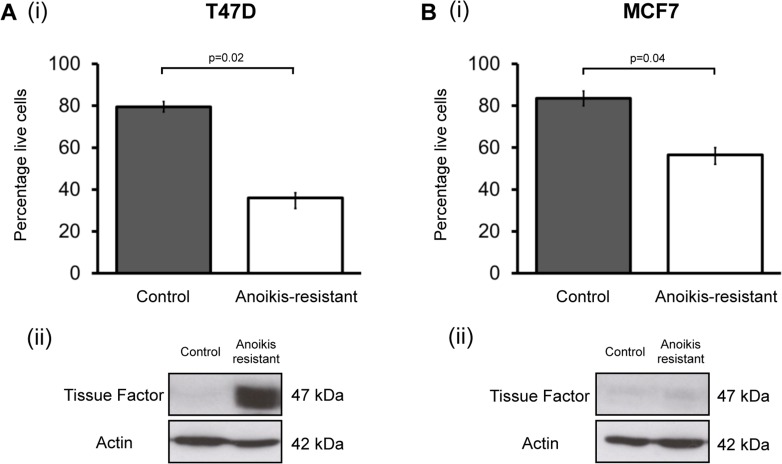
Tissue Factor expression is increased in anoikis-resistant (cancer stem cell enriched) cells Percentage of T47D (Ai) and MCF7 (Bi) breast cancer cells alive after 16 hours in normal adherent conditions (control) and non-adherent conditions (anoikis-resistant cells). Data is presented as percentage of live cells ± SEM (standard error of the mean) from 3 independent experiments. Protein lysates collected from these two populations underwent Western blotting to determine TF expression in control and anoikis-resistant populations. Representative Western blots are shown for (Aii) T47D and (Bii) MCF7. Actin expression was used as an approximate loading control. Western blots for each cell line are representative of at least 3 independent experiments.

Collection of anoikis-resistant cells 16 hours after seeding in non-adherent culture enriches for cells with high *in vivo* tumour formation ability [19, 20]. TF expression was determined in CSC enriched populations in T47D and MCF7 cell lines and compared to control. The percentage of T47D and MCF7 cells that survived non-adherent culture after 16 hours was significantly lower than cells plated in adherent conditions (Figure 1), as has previously been demonstrated [20]. TF expression (Western blotting) was compared in the adherent and non-adherent populations after removal of dead cells. In the CSC-enriched anoikis-resistant T47D populations there is a marked upregulation of TF protein expression compared to barely detectable TF expression in the control population. In MCF7s, which also have low TF expression, there is no apparent change in TF expression in the anoikis-resistant population compared to control (Figure 1).

The Aldefluor assay was used to identify a subpopulation of T47D cancer cells with increased ALDH1 enzymatic activity (ALDH1-high or Aldefluor-bright cells), as this is a recognised marker of increased CSC activity. TF expression was then assessed in the TF high population (which formed 1.7% of all cells). TF expression was higher (*p* = 0.05) on FACS analysis in the ALDH1-high population compared to the ALDH-low population, demonstrating increased TF expression in T47D cells with high CSC activity (Supplementary Figure 1).

### Cancer stem cell activity is increased in cells sorted for Tissue Factor expression

**Figure 2:**
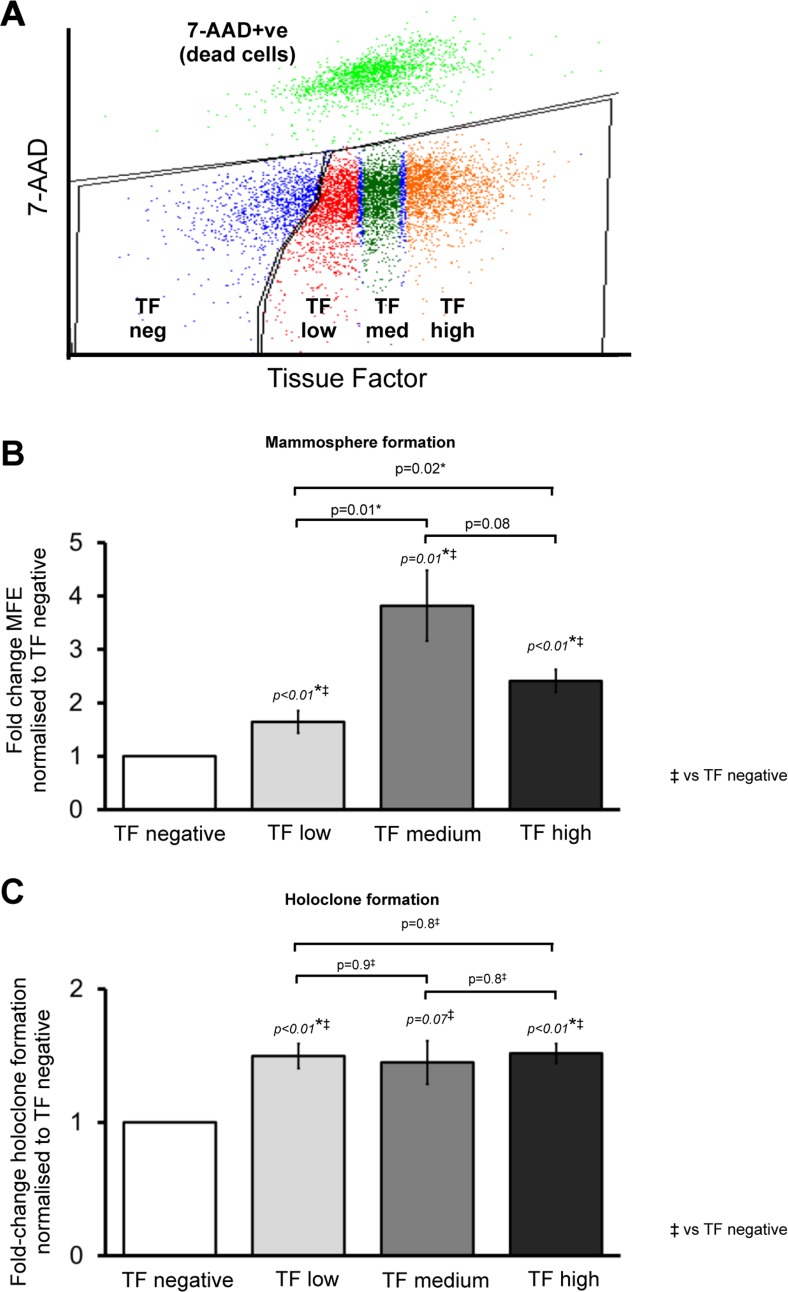
Cancer stem cell activity is increased in higher TF expressing breast cancer cells Representative FACS (flow cytometry) plot showing live T47D cells (identified using dead-cell marker 7-AAD) sorted into TF negative and TF positive populations that were further divided into TF low, TF medium and TF high expressing groups based on TF cell surface expression (**A**). Mammosphere forming efficiency (**B**) and holoclone colony formation (**C**) were determined in T47D cells sorted according to TF expression. Data is represented as fold change in mammosphere forming efficiency (MFE) or holoclone colony formation ± SEM normalised to control from at least 3 independent experiments. *p<0.05.

As TF expression was increased in CSC-enriched T47D cells, we hypothesised that CSC activity would be upregulated in cells sorted for TF expression (compared to TF negative cells). FACS sorting was used to sort T47D cells into TF negative cells and TF positive cells, the latter divided into tertiles based on the baseline fluorescence established using an isotype control antibody. The final four populations were TF negative, TF low, TF medium and TF high (Figure 2A).

Mammosphere forming efficiency (MFE) was increased (*p* < 0.01) in all three TF expressing populations (TF low, TF medium and TF high) compared to TF negative cells (Figure 2B). TF-low T47D cells demonstrated a 1.6 fold increase in MFE compared to TF-negative cells with higher increases see in TF-medium (3.8-fold) and TF-high (2.4-fold). When comparing the three TF expressing populations, MFE was higher in TF medium and TF high cells compared to TF low cells (*p* < 0.05) with TF medium cells having higher MFE than TF high (*p* = 0.08). Similar to mammosphere formation, holoclone colony formation increased in all three TF expressing populations compared to TF negative. No difference was seen, however, in holoclone formation between the TF-low, TF-medium and TF-high cells (Figure 2C).

### Forced Tissue Factor overexpression increases breast cancer stem cell activity

**Figure 3:**
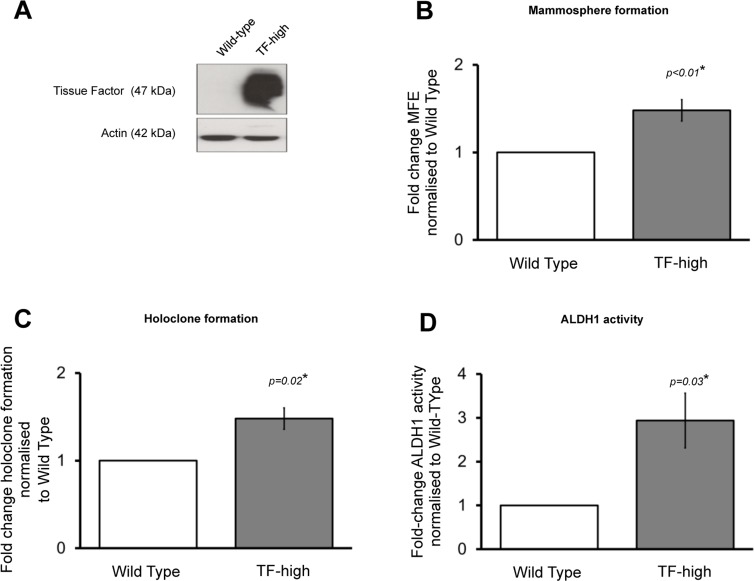
Forced TF overexpression increases breast cancer stem cell activity Western blot confirming increased TF expression in MCF7 TF overexpressing cells compared to Wild-type MCF7s (**A**). Mammosphere forming efficiency (**B**), holoclone colony formation (**C**) and percentage of ALDH1-high cells (**D**) was determined in TF-overexpressing and Wild-type MCF7 cells. Data is represented as fold change in mammosphere forming efficiency (MFE), holoclone colony formation or percentage ALDH1-high cells ± SEM normalised to control from at least 3 independent experiments. *p<0.05.

We next examined the effect of forced TF overexpression on CSC activity. MCF7 cells stably transfected to overexpress TF were used (Figure 3A). These cells were created using a genomic flippase recognition target (FRT) that allows insertion of a gene at a specific gene locus as previously described [21]. A ‘wild-type’ MCF7 cell was also constructed containing an empty vector at that FRT locus. Mammosphere formation, holoclone colony formation and ALDH1 activity was determined MCF7 TF overexpressing cells (TF-high) and compared to the wild-type control (WT). MFE was higher in the TF-high MCF7s (*p* < 0.01) compared to the WT control with the presence of TF increasing mammosphere formation by a factor of 1.5-fold (Figure 3B). Holoclone colony formation was also increased in the TF-high cells (*p* = 0.02) compared to WT (Figure 3C). ALDH1 activity was determined using the Aldefluor assay in both the TF-high and WT MCF7s. There was a 2.9 fold increase (*p* = 0.03) in the percentage of cells that had high ALDH1 activity in the TF-high compared to the WT cells (Figure 3D) further demonstrating that high TF expression is associated with increased CSC activity.

### Tissue Factor knockdown reduces cancer stem cell activity

**Figure 4:**
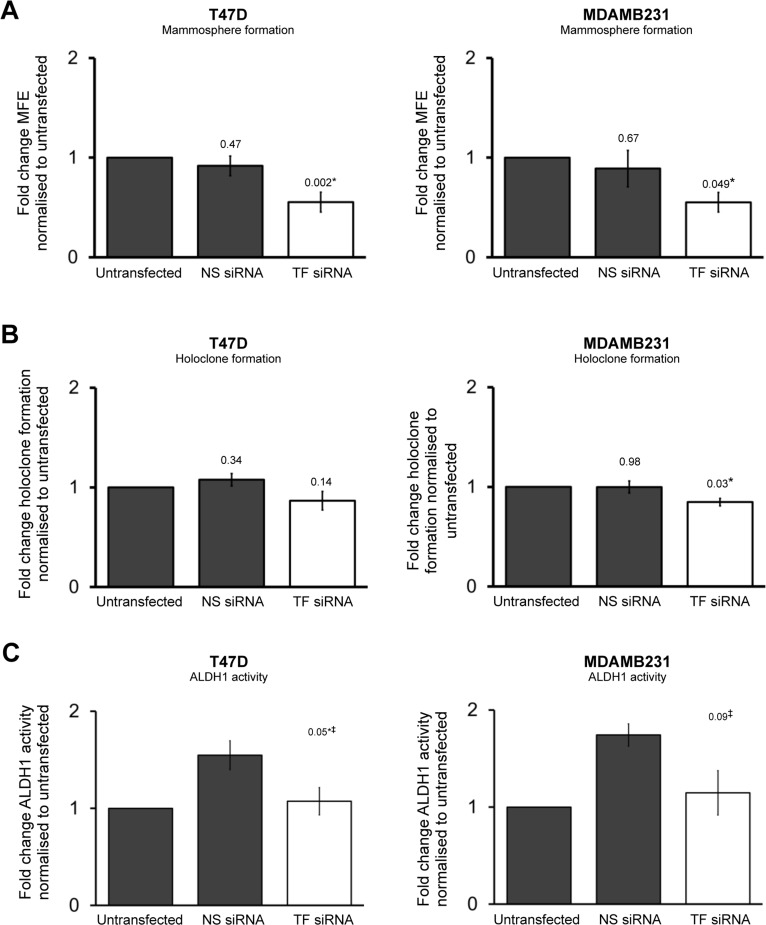
Transient TF knockdown reduces breast cancer stem cell activity Mammosphere forming efficiency (**A**), holoclone colony formation (**B**) and percentage of ALDH1-high cells (**C**) was determined in T47D and MDAMB231 cells in the presence of TF siRNA and compared to untransfected and control cells. Data is represented as fold change in mammosphere forming efficiency (**A**), holoclone colony formation (**B**) and ALDH1 activity (**C**) ± SEM normalised to control from at least 3 independent experiments. ‡Due to increase in fluorescence in presence of NS siRNA, comparison is made between fold change in ALDH1-high cells in TFsiRNA and NS siRNA and not untransfected control. *p<0.05.

We next examined the effect of TF knockdown on CSC activity in T47D and MDAMB231 cell lines. TF was knocked down with siRNA in the 231 and T47D cell lines (Supplementary Figure 2). MFE was reduced in T47D (*p* = 0.002) and MDAMB231 (*p* = 0.05) cell lines in the presence of TF siRNA compared to untransfected control (Figure 4A). Previous studies have demonstrated that TF siRNA reduces colony formation in lung adenocarcinoma cells plated in adherent conditions at low density [22], but no studies have examined the effects of TF knockdown on holoclone formation which acts a CSC activity marker. TF siRNA significantly reduced (*p* = 0.03) holoclone formation in the MDAMB231 cell line compared to control. No significant effect was seen, however, in holoclone formation in T47Ds when TF was knocked down (*p* = 0.1) (Figure 4B). Of note, colonies plated with MDAMB231 cells grew more quickly than T47D cell colonies and were usually fixed (with crystal violet dye) by day 7. T47D cell colonies usually require 10-14 days to grow to reach a size that is suitable for counting and TF knockdown appears to only persist for 6 to 7 days (Supplementary Figure 2) suggesting true effects of knockdown may have been missed in the T47D cells.

The effect of TF siRNA on ALDH1 activity was determined in T47D and MDAMB231 cancer cell lines. For each condition (untransfected control, NS siRNA, TF siRNA) cells underwent the Aldefluor assay 48 hours after transfection. Fluorescence in the FL1 FACS channel that is used for Aldefluor assay was found to increase in the presence of NS siRNA compared to untransfected control. On this basis, ALDH1 fluorescence in the presence of TF siRNA was compared to that of NS siRNA. ALDH1 activity (percentage of cells categorised as ALDH-high) was reduced in T47D cells (*p* = 0.05) and MDAB231 cells (*p* = 0.09) transfected with TF siRNA when compared to NS siRNA controls (Figure 4C).

### Tissue Factor agonist Factor VIIa increases mammosphere formation and ALDH1 activity

**Figure 5:**
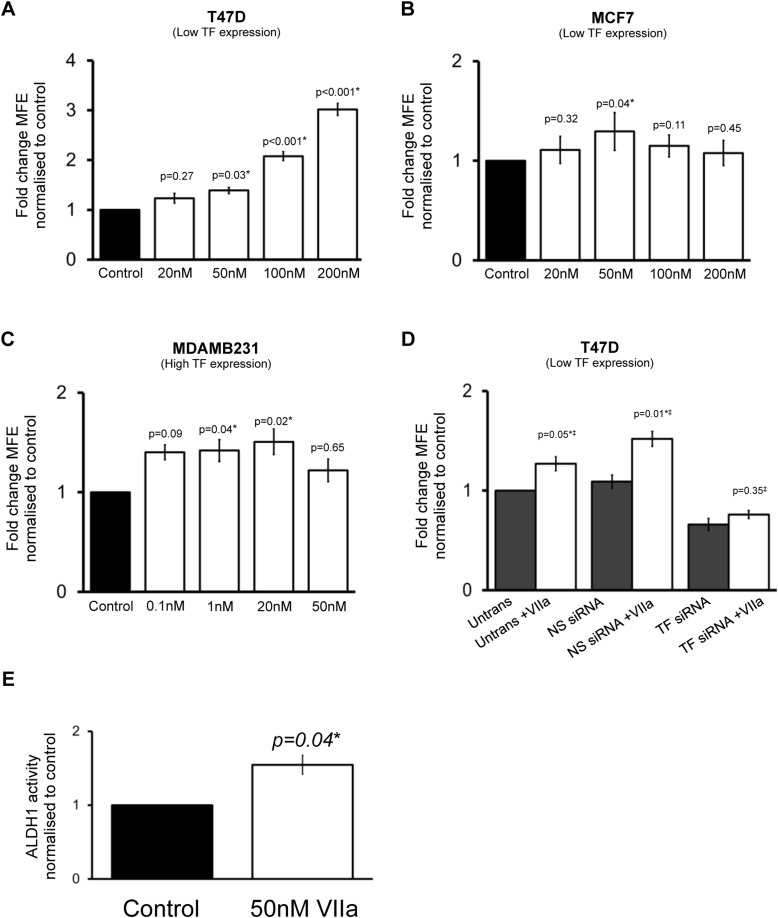
The effect of Factor VIIa on breast cancer stem cell activity Mammosphere forming efficiency (MFE) was determined in breast cancer cell lines T47D (**A**), MCF7 (**B**) and MDAMB231 (**C**) in the presence of increasing concentrations of Factor VIIa (FVIIa) compared to control (0.5% albumin). MFE was also determined in the presence of 50nM FVIIa in T47D following transfection with non-silencing (NS) siRNA or TF siRNA or in control (untransfected) conditions (**D**). ALDH1 activity was determined in T47D breast cancer cells incubated with FVIIa (**E**). Data in A to C is represented as fold change in MFE ± SEM normalised to control. Data in D is represented as fold change in MFE, ± SEM normalised to respective control (without FVIIa). Data in E is represented as fold change in ALDH1-high cells ± SEM normalised to control. ‡ Comparison is made with respective control (without FVIIa). Data is from at least 3 independent experiments. *p<0.05.

The clotting protein Factor VIIa (FVIIa) acts as a natural ligand for TF and is essential for TF's haemostatic and intracellular signalling functions [23]. FVIIa promotes apoptosis and anoikis resistance in TF expressing cells, so we hypothesised that it would increase mammosphere formation [14]. The effect of FVIIa on mammosphere formation was determined in T47D, MCF7 and MDAMB231 cells. Based on previous data [24–26], initial concentrations of between 1 and 100nM of FVIIa were used for mammosphere assay experiments. Preliminary experiments indicated that higher concentrations of FVIIa had effects on MFE in low TF expressing cells (e.g. T47D) and lower concentrations had effects in high TF expressing MDAMB231s. In the low TF-expressing T47D cell line, FVIIa produces a dose dependant increase in MFE compared to control. While 20nM of FVIIa did not increase MFE (*p* = 0.27), 50nm (*p* = 0.03) and 100nM (*p* < 0.001) significantly increased MFE with an almost 3-fold increase in MFE seen with 200nM of FVIIa compared to control (Figure 5A). MCF7s have similar TF expression to T47Ds however the effects of FVIIa on MFE were not as evident. MCF7s demonstrated an increase in MFE with 50nM FVIIa (Figure 5B).

In the high TF expressing MDAMB231 cell line, lower concentrations of FVIIa (0.1nM, 1nM, 20nM) appeared to have dose dependent effect of increasing MFE compared to control with 20nM of FVIIa causing a 1.5-fold increase in MFE compared to control. No effect on MFE was seen with 50nM of FVIIa (Figure 5C). The variation in response between cell lines may be due to variability of downstream pathway regulation, which could have implications in the clinical setting for different breast cancer subtypes. The variability in lack of response to higher doses may reflect variability in toxic effects of FVIIa, particularly at higher concentrations.

To confirm that FVIIa's actions on increasing mammosphere formation occurred through TF, FVIIa was incubated with T47D cells in the presence of TF siRNA. A single concentration of FVIIa (50nM) was added to transfected cells and then cells were seeded onto non-adherent plates 24 hours after transfection. In untransfected T47D cells and in cells transfected with NS siRNA, there was a significant increase (*p* < 0.05) in MFE in the presence of FVIIa as expected. FVIIa had no effect on MFE in cells lacking TF (TF siRNA) demonstrating that TF is required for the effect of FVIIa on mammosphere formation (Figure 5D), thereby supporting the hypothesis that FVIIa increases MFE through its receptor TF.

We next examined FVIIa's effects on CSC activity as measured by holoclone colony formation and ALDH1 activity. Holoclone colony formation was not increased in the presence FVIIa in MDAMB231 or T47D cells. However, in T47D cells the percentage of ALDH1-high cells was more than 1.5 times greater (*p* = 0.04) in T47D cells incubated with 50nm FVIIa compared to control (Figure 5E) providing further overall support to the hypothesis that FVIIa increases CSC activity.

## DISCUSSION

In this study we utilised established CSC activity assays to demonstrate a putative role for TF in regulating cancer stem cells (CSCs) *in vitro*. Two CSC-enrichment assays were used to demonstrate TF upregulation in T47D-derived CSCs. Anoikis-resistant T47D and MCF7 cells have previously been demonstrated to enrich for cells with increased *in vivo* tumour forming ability, the gold standard for CSC activity [19, 27]. In T47D cells (but not MCF7 cells), anoikis resistant cells showed increased TF expression compared to cells grown in adherent culture. Similarly, T47D cells with high ALDH1 activity were shown to have increased TF expression compared to the ALDH1-low population. Although only a small percentage of T47D cells demonstrated high ALDH1 activity, this subset of cells have been shown to correlate with *in vivo* tumour forming capacity in this cell line [28]. This finding is similar to that of Milsom's who demonstrated that CD133 positive A431 cells (which have increased CSC activity) had a 6-fold increase in TF activity compared to CD133 negative cells [18].

We next demonstrated an association between high TF expression and increased CSC activity. When sorted using flow cytometry, TF-expressing T47D cells demonstrated increased mammosphere formation and holoclone colony formation compared to TF negative cells. There was no clear positive correlation between greater TF expression and increased CSC activity in the TF expressing cells suggesting that the presence of TF is an important factor in increasing CSC activity and that at very high levels TF has inhibitory effects on proliferation and self-renewal. The loss of CSC activity in very high TF expressing cells may reflect a simultaneous loss of Endothelial Protein Receptor C (EPRC) cell expression. EPRC is a co-receptor in the anticoagulation pathway, with cell expression inversely related to TF expression [29] and EPRC expression associated with markedly increased CSC activity [29]. The loss of CSC activity in high TF expressing cells in our study may reflect the almost complete loss of EPRC expressing CSCs that is not outweighed by the gain in TF expressing CSCs. It is particularly interesting that both EPRC in the anticoagulation pathway and thrombin, a factor downstream of TF in the coagulation cascade mediate their signalling *via* PAR1, suggesting a balance of these two pathways may be critical in CSC activity.

MCF7 TF-overexpressing cells also demonstrated increased CSC activity compared to Wild-type MCF7s as measured by all three assays. Conversely, transient knockdown of TF inhibited CSC activity in two breast cancer cell lines. Mammosphere formation was significantly inhibited by TF siRNA in the low TF-expressing T47D and high TF-expressing MDAMB231 cell lines as well as reducing ALDH1 activity in both cell lines. This confirms, for the first time, the association between TF expression and CSC activity.

A dose-dependent increase in primary MFE was demonstrated in the presence FVIIa in T47D and MDAMB231 cells. In all three cell lines, FVIIa increased MFE. This was dose-dependent, most markedly in the T47D and MDAMB231 cell lines. However, the maximal efficacious dose varied between cell lines, with the low TF-expressing cells requiring higher FVIIa dose to increase MFE. This may be due to variations in TF:FVIIa complex formation or variability in downstream pathway regulation. TF is widely expressed in normal and disease states yet not all cell types respond to FVIIa stimulation [30]. In T47D cells, we showed that the effect of FVIIa on MFE was specific to TF as transient knockdown of TF in T47D abrogated the effect of FVIIa on MFE. FVIIa also increased ALDH1 activity in T47D cells further supporting the basis for the hypothesis that the TF ligand FVIIa increases CSC activity.

TF has been indirectly linked to transcriptional changes in proteins linked to CSC activity. This includes the pro-apoptotic protein MFG-E8 [31] and the transcription factor Nanog which is well described in CSC function [32]. FVIIa in conjunction with the PAR2 receptor activates the Mitogen activated protein kinases (MAPK) pathways such as p44/p42 as well as the PI3K/AKT pathway. Both pathways promote pro-malignant gene expression (such as cell proliferation and differentiation) but also play a role in cancer stem cell function [33]. Inhibition of both MAPK and PI3K/AKT pathways in colorectal cancer cells reduced clonogenic activity of CD133^+^ cells (a CSC marker) [34] while a selective PI3K inhibitor reduced *in vivo* tumour forming capacity of breast cancer cells [35]. FVIIa mediates its anti-apoptotic and anti-anoikis effects and *via* the PI3K and MAPK pathways [14, 36] suggesting the possibility that both pathways mediate the effects of TF/FVIIa signalling on cancer stem cell activity. PI3K and AKT activity is also linked to EGFR-dependant TF upregulation [37] that is associated with induction of epithelial to mesenchymal transition [15], a key feature of cancer stem cell activity [17]. Although exogenous Factor VIIa was only added to one set of experiments in this paper, exogenous production of FVIIa and subsequent association with surface TF has been demonstrated in ovarian cancer cells [38] suggesting the possibility of similar mechanisms in breast cancer cell lines. Interestingly, EGFR also upregulates ectopic production of FVIIa [39]. Although not applicable to this *in vitro* model, exogenous sources of FVIIa may also include cancer associated cells such as tumour associated macrophages [29]. TF upregulation in cancer cells contributes to macrophage recruitment [40] and these macrophages have been shown to facilitate conversion of dormant cancer cells to a tumorigenic (CSC-like) state [41].

There is marked genotypic heterogeneity between breast cell lines, as there are in clinical breast cancer. Gene expression profiling has identified five distinct phenotypes based on gene expression profiles. These overlap considerably with histological classifications based on expression of the ER, PR and HER2 receptors [42]. These classifications show reasonable overlap with commonly used cancer cell lines [43]. Recent classifications using genomic, transcriptional, translational data have further divided breast cancers into ten subtypes [44]. Despite both MCF7 and T47D cell lines having similar molecular phenotypes (Luminal-A-like, ER/PR positive) and both low-TF expressing, there was some variation in their relations between TF and CSC activity, for example in response to exogenous FVIIa. This may reflect genetic and epigenetic differences between cell lines, which are likely to also occur in the clinical setting of different breast cancer subtypes. For example, TF expression and/or function is known to be modulated by EGFR [15], the RAF-ERK pathway [45] and the k-ras/p53 pathway [12] all of which may vary between different classifications of breast cancer subtypes.

Although well described and reproducible, the cell-line based *in vitro* assays used in this paper can only act as surrogate markers for *in vivo* CSC activity and each has its limitations. Cell survival in adherent-free culture (anoikis resistance) is an important but not exclusive feature of CSCs and this needs to be considered when interpreting the association between anoikis resistance and TF expression. However, anoikis resistant cells derived from both T47D and MCF7 cells do enrich for cells with high mammosphere forming efficiency and increased *in vivo* tumour forming capacity (the latter the gold standard CSC assay) compared to control cells [19, 27]. Similarly, T47D cells with high ALDH1 activity correlate with *in vivo* tumour forming capacity [28]. A further limitation is that the cell-line derived CSC populations differ from breast cancer derived CSCs due to a greater accumulation of mutations and transformations. This can be addressed by assessing CSC activity of invasive or metastatic breast cancer derived cells ex-vivo using the mammosphere assay as previously described [46].

In this paper we demonstrate increased CSC activity correlating to TF expression. It should be noted that TF expression does not per se correlate with TF procoagulant activity [47]. In fact, the cancer promoting effects of TF are mediated through both procoagulant and coagulation-independent mechanisms [23]. Milsom and colleagues showed that CD133^+^-enriched A549 cells displayed increased TF procoagulant activity [18]. However the extent to which TF's effects on CSC biology are mediated via procoagulant or coagulation-independent pathways has yet to be elucidated. This has particular relevance in the clinical setting where anticoagulant therapies may have potential anti CSC activity. Moreover, identifying a role for TF signalling in CSC maintenance may enable CSC targeting without compromising normal haemostasis.

Trials involving TF inhibitors are currently underway in metastatic cancer and are due to report soon [48] [49]. A recently opened window of opportunity randomized controlled trial of Rivaroxaban in early breast cancer patients is exploring inhibition of the TF cofactor FXa in the oestrogen receptor negative subgroup of breast cancers, with biological endpoints including CSC activity. The success of anti-HER2 therapy in improving disease-free and overall survival may be mediated by its effect on CSC activity [50], highlighting the potential impact of CSCs as a therapeutic target.

In summary, we show that TF drives CSC activity. Targeting TF may be a novel means of treating breast cancer and reducing breast cancer recurrence.

## MATERIALS AND METHODS

### Cell lines and cell culture

Tissue Factor (TF) expressing human breast cancer cell lines T47D, MDAMB231 and MCF7 were purchased from the American Type Culture Collection (ATCC) and verified as mycoplasma free. MCF7 cells stably transfected to overexpress TF and matched empty-vector control were provided Dr Henri Versteeg from Leiden University Medical Centre (co-author). Cell lines were maintained in adherent culture conditions at 37oC at atmospheric pressure in 5% (v/v) carbon dioxide/air. MCF7 and T47D cells lines were cultured in complete DMEM medium (DMEM/10%foetal calf serum/2 mM L-glutamine/PenStrep) and the MDAMB-231 cell line was cultured in complete RPMI-1640 medium (RPMI/10% FCS/1% Sodium pyruvate/2 mM L-glutamine/PenStrep).

### Collecting anoikis-resistant cells

As previously described [27, 51], a single-cell suspension of disaggregated monolayer cells was plated at 1000 cells/cm^2^ into twenty poly-HEMA coated 225cm^2^ flasks (producing non-adherent conditions) and maintained at 37°C for 16 hours in mammosphere medium. Medium was collected and cells isolated by centrifugation (580g for 2 minutes) and underwent dead cell removal with MACS^©^ dead cell removal kit. Cells were centrifuged and the pellet underwent lysis using standard protein lysis buffer and stored at -20°C until use. A matched sample of cells from the same passage were plated in normal adherent conditions also at 1000 cells/cm2.

### Mammosphere culture

Mammosphere culture was performed as previously described [8]. A single cell suspension was seeded at 500/cm^2^ in poly-HEMA (Sigma) coated cells for 5 days in mammosphere media (DMEM/F12 phenol red free, 20ng/ml hEGF, B27) in normal culture conditions. Mammosphere forming efficiency (MFE) was calculated by dividing the number of mammospheres > 50μm in size formed divided by the number of single cells plated per well plated expressed as a percentage.

### Holoclone colony assay

As previously described [27], a single cell suspension was seeded at 50 cells/cm^2^ in *adherent* conditions in 6-well plates. Cells were grown for a maximum of 14 days. Colonies were fixed and stained with 1% (w/v) crystal violet/70% (v/v) ethanol after 14 days or earlier if colonies were close to meeting each other. Individual colonies (holoclone, paraclone and meroclone) were identified and the number of holoclone colonies was counted and expressed as a percentage of original cells plated.

### Flow cytometric analysis

As previously described [52], cells were suspended in PBS at a concentration of 1.2×10^7^ cells/ml and incubated with mouse anti-TF antibody (ADG4508, Sekisui, MA) 1:25 or isotype mouse IgG antibody (AB18447, Abcam, SF) 1:50 for 20 minutes at RT. After PBS washes, cells were incubated for 20 minutes with APC-linked goat anti-mouse-IgG antibody (A865, Invitrogen) at RT before resuspending in PBS. The Aldefluor ^TM^ assay kit (Stem Cell Technologies, Vancouver, Canada) was used to measure ALDH1 enzymatic activity according to manufacturer's instructions. As previously described [5], cells were resuspended at a concentration of 1×10^6^ cells/ml. 5μl of Aldefluor reagent was added and 500 μl was immediately transferred to a tube containing the ALDH1 inhibitor DEAB. Samples were incubated at 37°C for 30 minutes before centrifugation and collection of cell pellet. Baseline fluorescence was established by inhibiting ALDH1 activity with DEAB and used to generate a gate (top 0.1%) to identify ALDH1-high cells that have not been incubated with DEAB. Fluorescence was measured using FACSCalibur (BD Biosciences) and analysed using FlowJo Version 10.1.1.

### Sorting for TF expression

Cells were incubated with an anti-TF and anti-mouse APC linked secondary antibody as per cell surface expression protocol. In the final step, cells were resuspended in Hank's buffered saline solution and 5ul of 7-AAD was added per 1 × 10^6^ cells to identify dead cells. Cells were sorted using the FACS Aria (Becton Dickinson).

### Tissue Factor siRNA and transfection

A SMARTpool of four siRNAs targeting TF (L-004462-00-0000, Dharmacon, UK) and a mock scrambled siRNA (D-001810-10-05, Dharmacon, UK ) were used. Cells were plated at 5 × 106 cells per 6cm plate and transfection performed using Lipofectamine 2000 according to manufacturer's instructions. Cells were harvested at 24 or 48 hours post-transfection according to the experiment.

### Treatment of cells with recombinant Factor VIIa for mammosphere and FACS assays

Recombinant FVIIa was diluted with 0.5% albumin. Albumin was used for control conditions. Monolayer cells were incubated appropriate concentrations of FVIIa (which varied from 0.1nM to 200nM) for 24 or 48 hours depending on the experiment. This was based on concentrations used in previous publications [24–26].

### Western blotting

Protein content was measured using Pierce^©^ BCA Protein Assay Kit (Thermo Scientific, Rockford, IL). Protein were separated by SDS-PAGE under reducing conditions and transferred to Hyobond™-ECL Nitrocellulose membrane using Mini Trans-Blot Cell (Bio-Rad, Hertfordshire, UK). Primary antibodies were TF (ADG4508, Sekisui, MA) 1:250 and actin (SC-1616, Santa Cruz, CA) 1:500. HRP secondary reaction was catalysed using the Amersham™ ECL plus Western Blotting kit (GE Healthcare UK, Buckinghamshire).

### Statistical analysis

Data are represented as mean ± SE taken over a minimum of three independent experiments, unless otherwise stated. Statistical significance was measured using parametric testing, assuming equal variance.

## SUPPLEMENTARY MATERIALS FIGURES


