# Supplementary material for: Tissue Factor promotes breast cancer stem cell activity in vitro
Source: Oncotarget. 2016 Dec 13;8(16):25915–27. doi: 10.18632/oncotarget.13928 (PMC5432226; doi:10.18632/oncotarget.13928)
Supplement: Supplementary file 1 [file oncotarget-08-25915-s001.pdf]

Tissue Factor promotes breast cancer stem cell activity *in vitro*

Supplementary Material

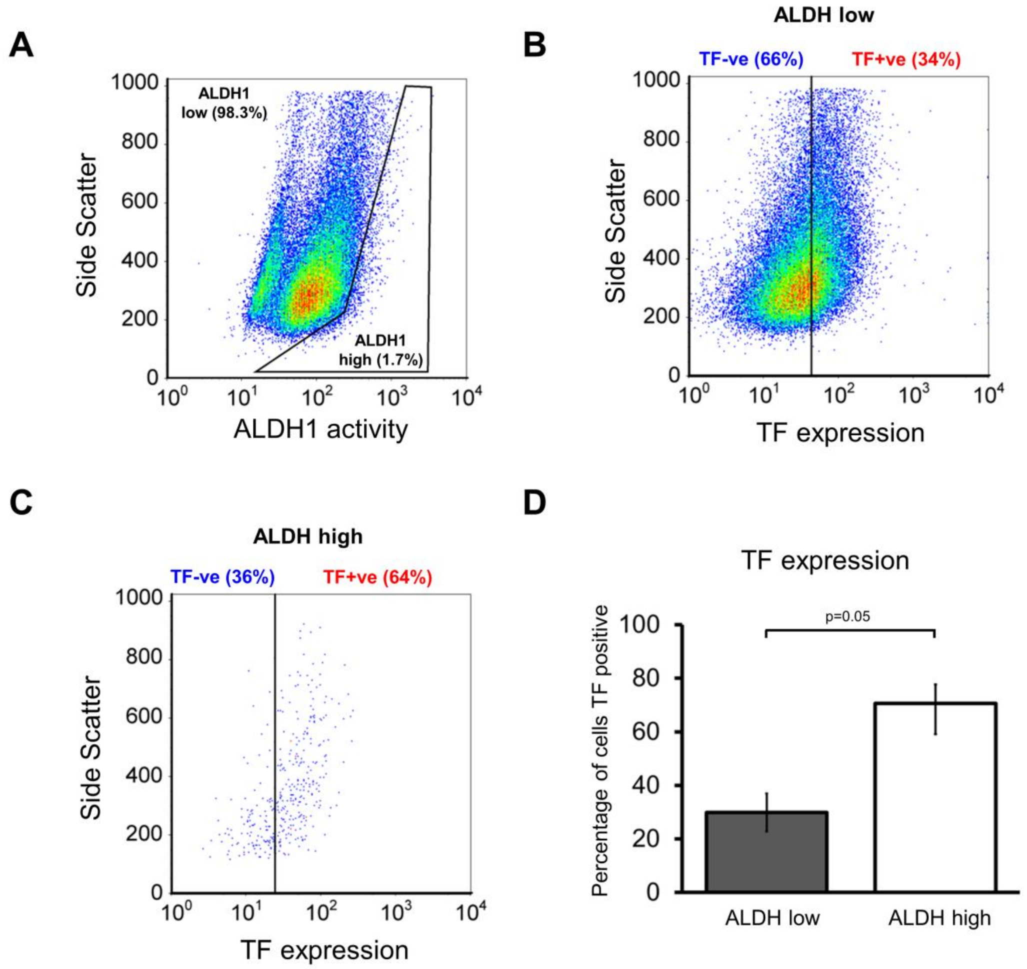

Supplementary Figure 1: Tissue Factor expression is increased in ALDH1-high (cancer stem cell enriched) cells

Representative plots of FACS analysis of ALDH1 activity and TF expression in T47D cells. ALDH1-high cells form 1.7% of the total T47D population (A). Co-expression of TF was determined in the ALDH1-low (B) and ALDH1-high T47D cells (C) and the percentage of TF expressing cells in the ALDH1-low and ALDH1-high cells was compared (D). Data is presented as percentage of TF positive cells ± SEM from 3 independent experiments

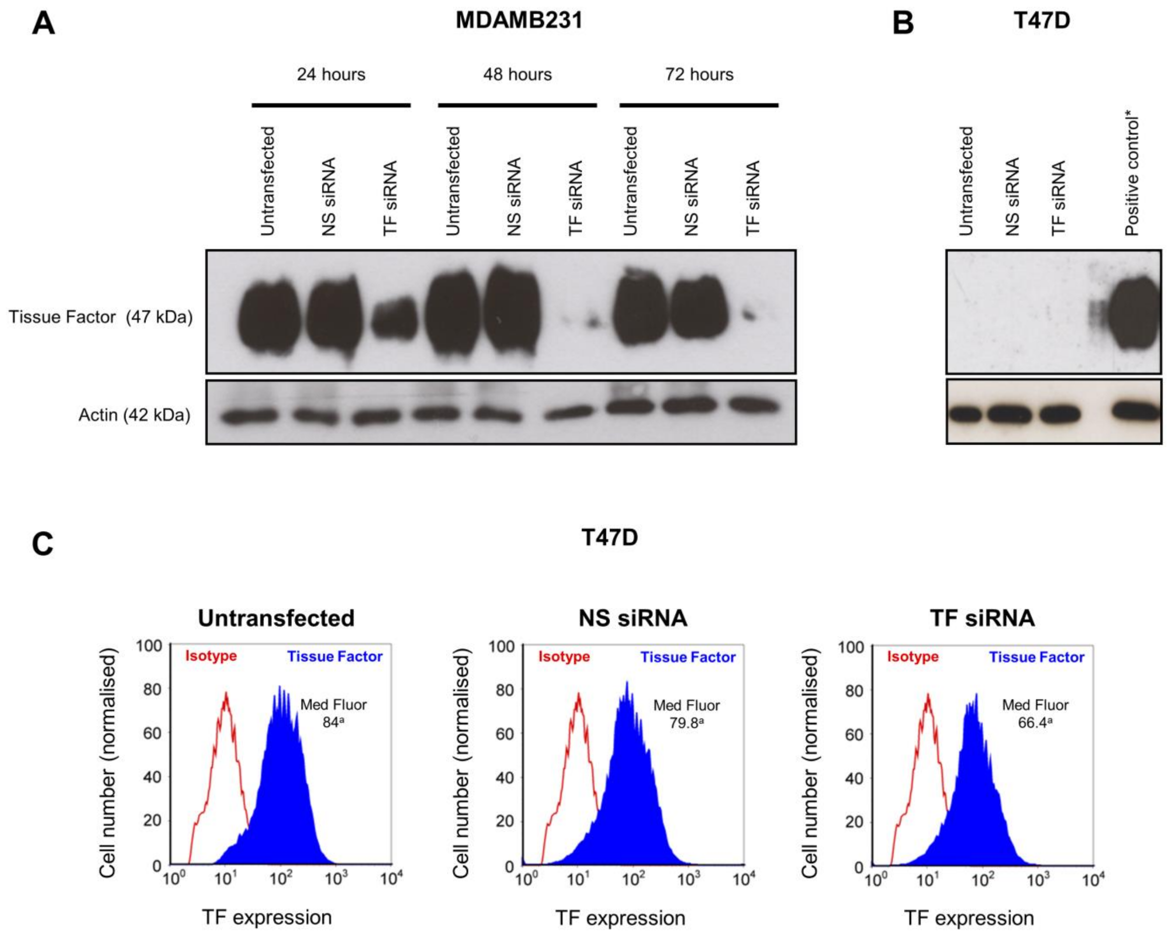

Supplementary Figure 2: Confirmation of TF knockdown in MDAMB231 and T47D cell lines
Western blot confirming TF knockdown at 24, 48 and 72 hours following transfection in MDAMB231 cells (A). TF expression is barely detectable in T47D cells before and after transfection (B), therefore flow cytometry is used to confirm reduction in TF expression in cells transfected with TF siRNA (C).
